# Heuristic Path Search and Multi-Attribute Decision-Making-Based Routing Method for Vehicular Safety Messages

**DOI:** 10.3390/s23239506

**Published:** 2023-11-29

**Authors:** Lei Nie, Junjie Zhang, Haizhou Bao, Yiming Huo

**Affiliations:** 1 School of Computer Science and Technology, Wuhan University of Science and Technology, Wuhan 430065, China; zhangjunjie0726@126.com (J.Z.); baohaizhou@wust.edu.cn (H.B.); 2 Hubei Province Key Laboratory of Intelligent Information Processing and Real-Time Industrial System, Wuhan University of Science and Technology, Wuhan 430065, China; 3 Department of Electrical and Computer Engineering, University of Victoria, Victoria, BC V8P 5C2, Canada; yhuo@ieee.org

**Keywords:** heuristic path search, multi-attribute decision-making, safety message transmission, routing method, vehicular network

## Abstract

Efficient routing in urban vehicular networks is essential for timely and reliable safety message transmission, and the selection of paths and relays greatly affects the quality of routing. However, existing routing methods usually face difficulty in finding the globally optimal transmission path due to their greedy search strategies or the lack of effective ways to accurately evaluate relay performance in intricate traffic scenarios. Therefore, we present a vehicular safety message routing method based on heuristic path search and multi-attribute decision-making (HMDR). Initially, HMDR utilizes a heuristic path search, focusing on road section connectivity, to pinpoint the most favorable routing path. Subsequently, it employs a multi-attribute decision-making (MADM) technique to evaluate candidate relay performance. The subjective and objective weights of the candidate relays are determined using ordinal relationship analysis and the Criteria Importance Through Intercriteria Correlation (CRITIC) weighting methods, respectively. Finally, the comprehensive utility values of the candidate relays are calculated in combination with the link time and the optimal relay is selected. In summary, the proposed HMDR method is capable of selecting the globally optimal transmission path, and it comprehensively considers multiple metrics and their relationships when evaluating relays, which is conducive to finding the optimal relay. The experimental results show that even if the path length is long, the proposed HMDR method gives preference to the path with better connectivity, resulting in a shorter total transmission delay for safety messages; in addition, HMDR demonstrates faster propagation speed than the other evaluated methods while ensuring better one-hop distance and one-hop delay. Therefore, it helps to improve the performance of vehicular safety message transmission in intricate traffic scenarios, thus providing timely data support for secure driving.

## 1. Introduction

Over the past few decades, the surge in urban vehicle numbers has intensified challenges in city transportation systems, notably in traffic safety and efficiency. The Vehicular Ad Hoc Network (VANET) has emerged as a cornerstone for Intelligent Transportation Systems (ITSs), offering a promising solution to these challenges [1]. VANET has garnered extensive research attention and has found applications in signal control [2,3], route optimization [4,5], cooperative tasks [6,7,8], and safety communication [9,10], among others. Particularly, VANET has significant research merit and practical value in safety communication. It paves the way for a streamlined and secure driving landscape, minimizing accidents and curtailing economic costs.

Owing to the dynamic nature of the VANET topology, vehicles often grapple with inconsistent communication links amongst themselves. Such instability can quickly escalate to network congestion, packet loss, and significant end-to-end delays. In crisis situations like traffic accidents, it is imperative that safety messages are dispatched promptly and reliably to the intended destination or vehicle [11,12]. The intricate urban traffic landscape further complicates matters; disjointed road sections can disrupt safety message transmission, and suboptimal message relays hamper swift message propagation. Thus, efficient VANET routing strategies should prioritize both path and relay selection to ensure the promptness and dependability of safety message delivery.

**Figure 1:**
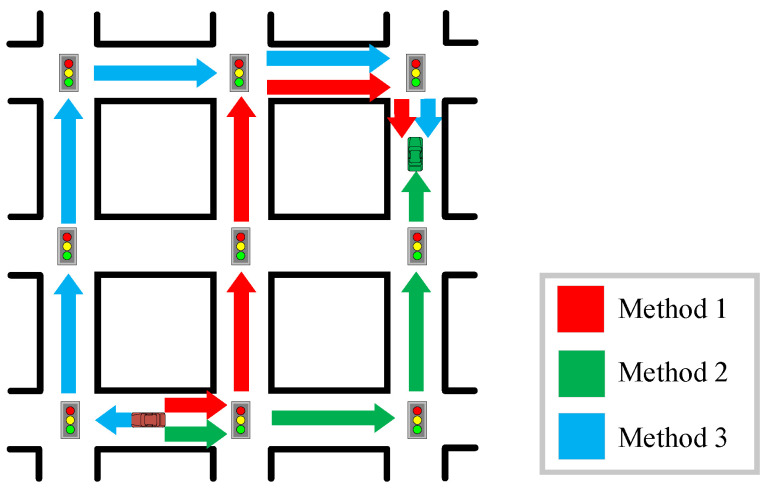
Examples of different path selection strategies.

As shown in Figure 1, three routing methods with different path selection strategies are presented in a vehicular safety message transmission scenario. Method 1 tends to find the shortest path, Method 2 tends to find a path with the highest connectivity, and Method 3 forms a path based on constantly selecting relays, which leads them to select different routing paths. In addition, even if the same paths are selected, the performance of the routing methods may vary due to different relay selection strategies.

For path selection, many current routing approaches tend to concentrate solely on identifying the next best intersection from a given point. This narrow focus can result in localized optimization issues. Regarding relay selection, these methods often prioritize one-hop distance or link quality, neglecting the interplay and significance of multiple evaluation metrics. This oversight complicates the accurate evaluation of relay performance in intricate traffic scenarios. Existing routing methods, therefore, face difficulty in determining a globally optimal transmission path or are unable to accurately assess relay performance, leading to poor performance in routing in intricate traffic scenarios, which affects timely data support for secure driving. It is, therefore, necessary to design an efficient routing method that takes into account the selection of globally optimal transmission paths and relays for timely and reliable transmission.

In this study, we design a heuristic path search and multi-attribute decision-making-based routing (HMDR) method for vehicular safety messages. Our main contributions are as follows:

1. For the identification of a global optimal transmission path, HMDR initiates by constructing a path tree model spanning from the destination intersections back to the source intersection. It then pinpoints the most efficient route through a heuristic path search that emphasizes road section connectivity;
2. To accurately evaluate the performance of candidate relays, the HDMR method employs a multi-attribute decision-making-based approach. This calculates the subjective and objective weights of candidate relays based on the ordinal relationship analysis method and the Criteria Importance Through Intercriteria Correlation (CRITIC) weighting method, respectively;
3. The innovative HMDR strategy synergistically blends path search with relay selection, equipping it to manage safety message transmission in multifaceted traffic scenarios. Our experimental results underscore the effectiveness and proficiency of HMDR in this domain.

The remainder of this paper is organized as follows: Section 2 reviews some related work. A detailed description of the proposed HDMR method is given in Section 3. The performance of the HDMR method is evaluated in Section 4. Finally, we conclude the study in Section 5.

## 2. Related Work

Existing VANET routing methods are mainly categorized into network-topology-based, cluster-based, and geographic-location-based types. Network-topology-based routing methods store the routing information of the network nodes in a routing table and periodically update the routing table to maintain its availability. Perkins et al. proposed a hop-by-hop Destination Sequenced Distance Vector (DSDV) routing method [13], wherein each node maintains a routing table to other nodes; the route with a large sequence number and a small number of hops is the optimal route, and the routing table is periodically updated network-wide or partially to maintain the effectiveness of communication. Since then, Perkins et al. proposed an Ad Hoc On-demand Distance Vector (AODV) routing method based on DSDV [14], wherein the source node initiates the route lookup process only when a packet is sent to the destination node. If a link is found to be broken, the node sends an “error” message for route repair. Kanani et al. built a collision-avoidance system using the DSDV routing protocol and successfully forwarded emergency messages over VANET [15]. The DSDV routing protocol is used to provide a reliable and efficient routing solution and maintains a routing table for each node. Malnar et al. proposed a Neighbourhood Density AODV (ND-AODV) routing method to reduce routing overhead in large-scale dynamic wireless ad hoc networks [16]. The proposed routing method uses an expected transmission count (ETX)-based metric called Power Light Reflection ETX (PLRE) instead of the hop count metric, which greatly improves the reliability of the AODV protocol. However, in a vehicular network environment where the network topology changes frequently, the network topology-based routing method tends to consume and waste network resources because it must constantly interact with packets to update the routing table.

Cluster-based routing approaches use hierarchical network organization by cluster head nodes in order to reduce network communication and, thus, network resource overhead. Knowing how to select cluster head nodes and optimize the data transmission path to balance the network load and reduce the network communication overhead are urgent optimization problems. Zhang et al. proposed a new algorithm—AODV-MEC—for AODV clustering based on an edge computing strategy [17], which considers vehicle node energy and travel speed to select stable multi-hop links. It solves the problem of resource bandwidth consumption and additional network delay caused by offloading computing tasks to the cloud core network and improves the routing efficiency in the vehicular network. Kandali et al. proposed a new cluster-based vehicular routing protocol that combines a modified k-means algorithm with a continuous Hopfield network and Maximum Stable Set Problem (KMRP) [18]; the cluster head is selected via the weight function according to the amount of free buffer space, the speed, and the node degree. The experimental results show that it performs better in highway vehicular environments. Raja proposed a perspective on road safety by adopting a routing protocol for hybrid VANET-WSN communication (PRAVN) [19]; the proposed routing protocol uses the Improved Water Wave Optimization (IWWO) algorithm for clustering, which minimizes energy consumption while maintaining balanced clusters to monitor bandwidth. However, knowing how to reasonably select cluster head and forwarding nodes and maintain the stability of the cluster structure is a common problem faced by cluster-based routing methods.

Location-based routing methods use the location information between vehicles to decide the relay of route transmission without consideration of route discovery, route maintenance, and network topology, which is more suitable for high-mobility vehicular networks. Karp et al. proposed a greedy peripheral stateless routing protocol named Greedy Perimeter Stateless Routing (GPSR) [20], which combines greedy routing with surface routing and uses surface routing to avoid local minima where greedy forwarding fails. However, due to the presence of obstacles, packets may travel through longer paths and result in higher latency. Zhang et al. proposed a Weight-based Path-aware Greedy Perimeter Stateless Routing (W-PAGPSR) protocol [21]. At the routing establishment stage, the proposed protocol integrates the node distance, reliable node density, cumulative communication duration, and node movement direction to evaluate the communication reliability of the nodes, and the next-hop node is selected using a greedy weight forwarding strategy. Chen et al. proposed an Artificial Spider Geographic Routing (ASGR) algorithm for urban environments [22]. From the perspective of bionics, a spider-web-based network topology is constructed; a connection quality model and transmission delay model are established to select the optimal path from all feasible paths; and, finally, a selective forwarding scheme is proposed based on the node motion and signal propagation characteristics. Rana et al. [23] proposed a novel routing model named Fuzzy-logic-based Multi-hop Directional Location Routing (FLMDLR) in VANET. FLMDLR selects outstanding next hops that help to establish a stable routing path from the source to the destination. In intricate traffic scenarios, the location-based routing method is currently the most reasonable and effective method.

The key to the safety message routing problem in vehicular network environments lies in knowing how to select appropriate routing paths and relays and is reflected in the transmission delay, speed, and coverage of safety messages. However, combined with the above description and summary, existing routing methods often fail to solve the key problem described above, which affects timely data support for secure driving. In this study, we intend to study and propose the method of combining path and relay selection, which integrates the effects of transmission paths and relay nodes on the quality of communication links and message propagation speed to adapt to intricate traffic scenarios.

## 3. Heuristic Path Search and Multi-Attribute Decision-Making-Based Routing Method

### 3.1. System Model and Assumptions

The system model and related assumptions are given in this section, and the meaning of the main variables frequently used in this paper are listed in Table 1.

**Figure 2:**
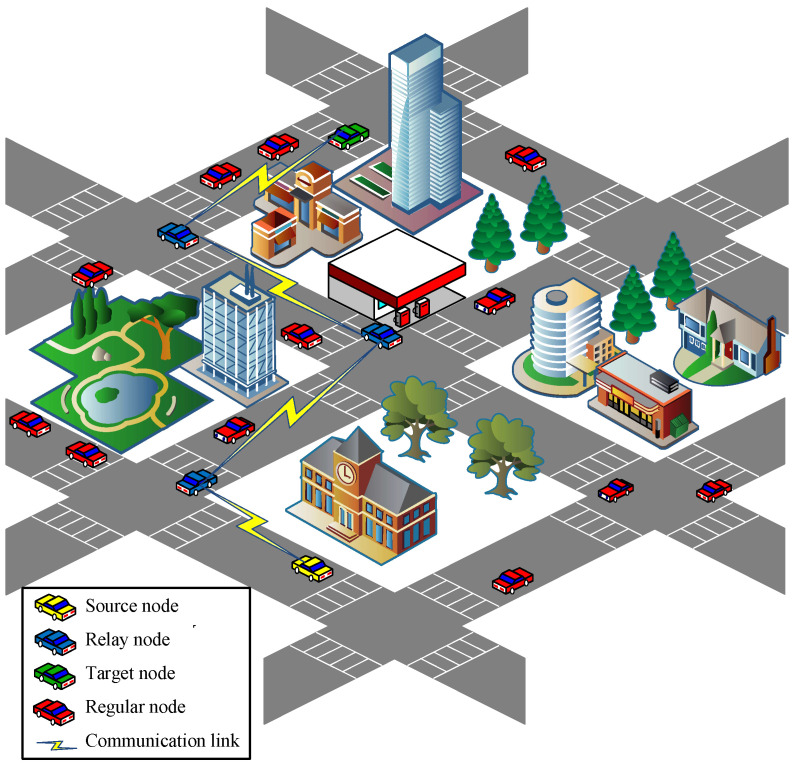
System model.

In our study, an urban vehicular network environment within a certain geographical range is analyzed as a whole. A system model of our study is shown in Figure 2, where vehicles are traveling in a multi-lane urban traffic scenario. The safety messages are generated via the yellow vehicle and transmitted to the green target vehicle using the blue vehicles as relays, while the other red vehicles are ordinary nodes that only receive safety messages. The system model satisfies the following assumptions:

1. Initially, the vehicles with Poisson distribution travel in the urban traffic scenario with multiple lanes in both directions;
2. Each vehicle is equipped with an On-board Unit (OBU) that utilizes a Dedicated Short-range Communication (DSRC) interface for Vehicle-to-vehicle (V2V) communication and obtains location information based on GPS and electronic map;
3. Each vehicle receives road section information from the equipped electronic map, as well as periodical Beacon messages (including vehicle ID, speed, position, timestamp, etc.) from its neighbors, and maintains a road section information table and a neighbor information table;
4. Both buildings and trees attenuate the RSS of safety messages, and each vehicle records the RSS values of monitored neighboring vehicles in the neighbor information table.

### 3.2. Overview of HDMR

**Figure 3:**
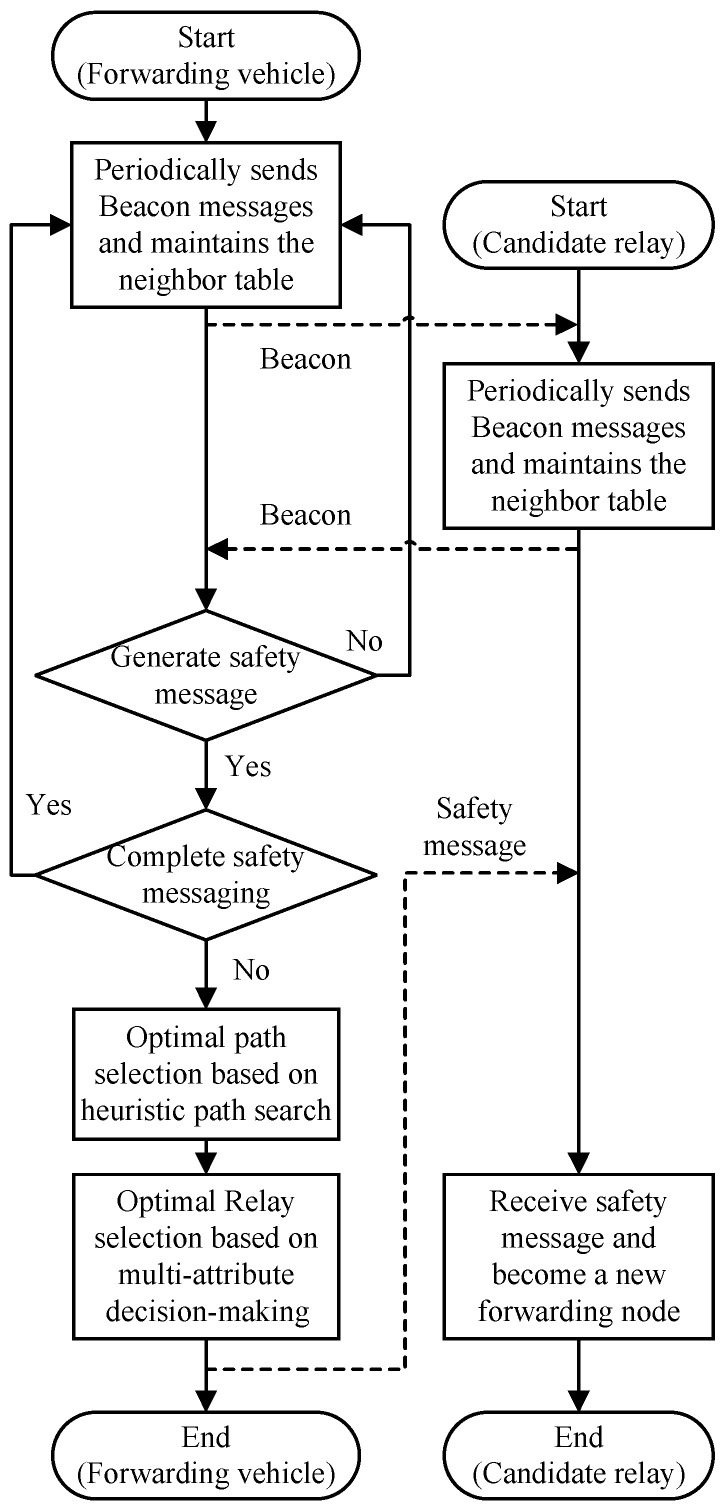
The main process of HDMR.

The main process of HMDR is shown in Figure 3. In a V2V communication-based vehicular network environment, each vehicle obtains information about relevant vehicles and road sections through periodic Beacon messages as well as in-vehicle GPS and electronic maps, while, at the same time, the current forwarding node of the safety message interacts with the candidate relays and selects the optimal relay by executing the HMDR method. In this process, the main problems that require a solution are searching for the optimal path and selecting the optimal relay, which are two important phases of HMDR.

In the optimal path search phase, the source and destination intersections are firstly determined based on the geographical location of the source node and the target node; then, the path model, based on the connectivity of road sections, is established according to road-section-related information, and the least costly path is finally selected as the optimal routing path.

In the optimal relay selection phase, the current forwarding vehicle first calculates the utility values of the four evaluation metrics of candidate delays by using corresponding utility functions and constructs the decision matrix. Then, it calculates the subjective and objective weights of the metrics of candidate relays by using ordinal relationship analysis and CRITIC weighting methods, respectively, and obtains the comprehensive utility values reflecting the performance of candidate relays. Finally, it calculates the node with the biggest comprehensive utility value combined with the link time as the next forwarding node of the safety message.

When a candidate relay is selected as the optimal relay and receives the safety message, it becomes a new forwarding node and continues to select a new relay based on HDMR. The above steps are repeated until the safety message reaches the destination after multi-hop forwarding.

### 3.3. Optimal Path Search

Intersections in urban environments are usually regarded as key nodes for finding routing paths. In the first optimal path search phase, the HMDR method determines the source and destination intersections based on the geographical locations of the source and target vehicles and constructs a path tree model to obtain all possible transmission paths; then, the connectivity value of each road section is calculated. Finally, the optimal path with the least cost is determined using a heuristic search method.

#### 3.3.1. Path Tree Model

In this subsection, we use a tree structure to search for routing paths, called the path tree model. The path tree stores the relationship between intersections in the form of an adjacency table and adopts the depth-first search method for traversing the routing paths. The traversal time complexity of a path tree is O(V+E), where *V* is the number of vertices (i.e., intersections) and *E* is the number of edges (i.e., road segments). As the number of intersections increases, the path tree still traverses all of them quickly. In addition, the path tree avoids path loops so that there are no duplicate segments in the routing path.

**Figure 4:**
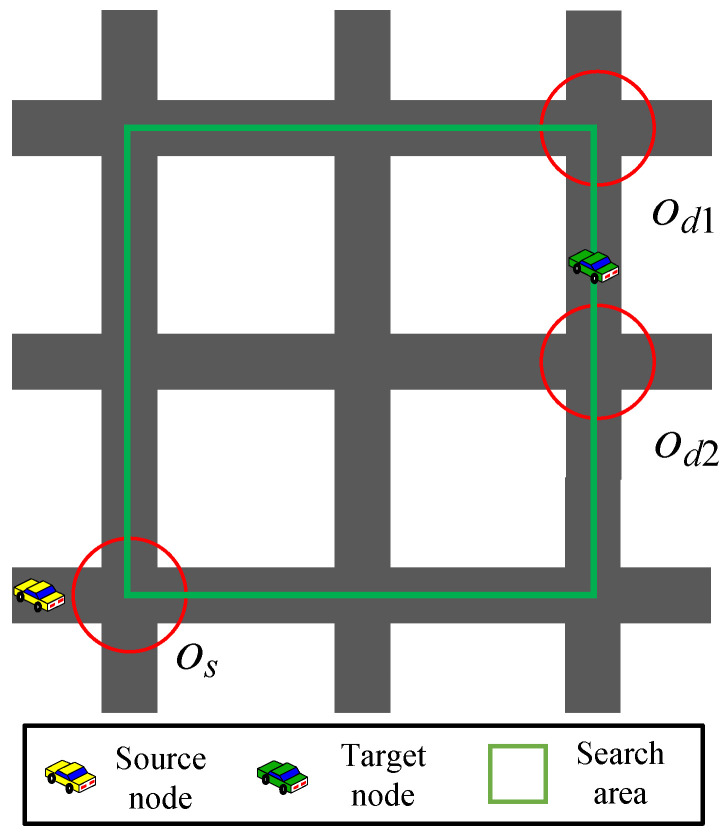
Example of an optimal path search area.

The selection of source and destination intersections is related to the geographic location of the source and target vehicles. If the source or target vehicle is located at an intersection, the intersection is considered the source or destination intersection; if the source vehicle is located at a road section, the intersection closer to the destination intersection is considered the source intersection; and if the target vehicle is located at a road section, the intersections at both ends of the road section are considered the destination intersections. As shown in Figure 4, after selecting the source intersection $o_{s}$ and the destination intersections $o_{d1}$ and $o_{d2}$, extension lines are drawn along the road direction from these intersections, and the maximum quadrilateral area formed by the intersection of the four extension lines is the search area of the optimal path.

**Figure 5:**
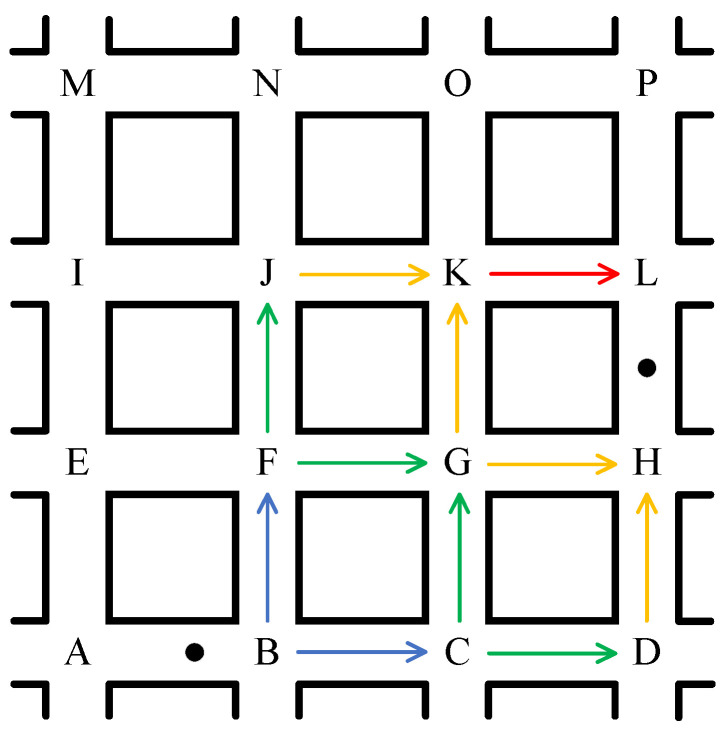
A case of a completed path model.

After determining the optimal path search area, the path model can be created by extending from the source node to the destination node. The source node is recorded as layer 0 nodes, its adjacent intersections as layer 1 nodes, the subsequent adjacent intersections as layer 2 nodes, etc., until the destination intersection is covered, and a completed path model is shown in Figure 5.

After completing the path model, path trees are constructed using the following rules:

1. Each path tree starts at a destination intersection and ends at the source intersection;
2. When an intersection is added to a path tree, it is removed from the set of intersections to which it belongs;
3. The children of an intersection in layer i+1 can only be its neighboring intersections in layer *i*;
4. The construction of all path trees is completed when the intersection set is NULL.

**Figure 6:**
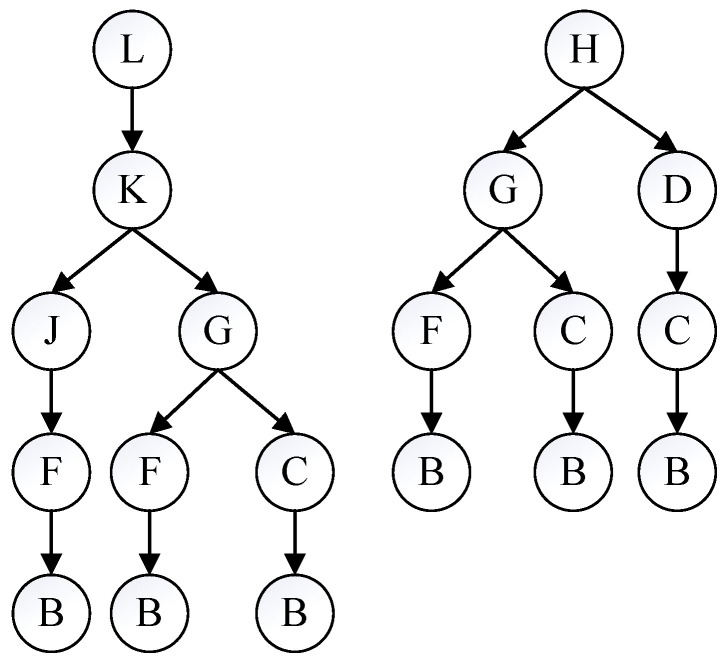
A case of constructing path trees.

According to the above rules, path trees with the destination intersection as the root are generated. As shown in Figure 5, the destination intersections are *L* and *H*. A depth-first traversal algorithm is used to find all available paths starting from the root and ending at the leaf nodes, the reverse paths of which are feasible paths from the destination to the source node, including LKJFB, LKGFB, LKGCB, HGFB, HGCB, and HDCB, as shown in Figure 6. In this way, it is possible to find the optimal one among these paths to satisfy specific requirements.

#### 3.3.2. Connectivity Analysis of Road Sections

In addition to the length of the routing path, the connectivity of the routing path, also known as the connectivity of the communication link, is an important factor that affects the message transmission delay, and since the routing path is composed of multiple road sections, it is necessary to analyze the connectivity of road sections in order to select a more reliable routing path.

In a V2V communication-based vehicular network environment, each vehicle obtains road and vehicle-related information based on an installed electronic map and receives periodic Beacon messages from its neighbors. Referring to the literature [24], the special density $\rho_{i,j}$ of candidate relays over the road section $l_{i,j}$ is defined and calculated as shown below:

(1) $$\rho_{ij}=\frac{R\cdot N_{ij}}{\left|l_{i,j}\right|},$$

where *R* is the communication radius of vehicles and $\left|l_{i,j}\right|$ and $N_{ij}$ are the length of $l_{ij}$ and the number of vehicles on $l_{ij}$, respectively.

According to the Poisson distribution, the probability of *k* vehicles occurring in the counting interval on $I_{ij}$ is denoted as $P_{ij}\left(k\right)$ as follows:

(2) $$P_{ij}\left(k\right)=\frac{\rho_{ij}^{k}\cdot e^{-\rho_{ij}}}{k!}.$$

To calculate the connectivity of $l_{ij}$, the road section is divided into $2\left|l_{ij}\right|/R$ sub-segments of length R/2. The probability that there is at least one vehicle on a sub-segment is shown in Equation (3):

(3) $$P_{ij}\left(k>0\right)=1-P_{ij}\left(0\right)=1-e^{-\rho_{i,j}}.$$

The fact that a road section is connected indicates that all its sub-sections are connected, i.e., there is at least one vehicle in each sub-section; thus, the connectivity of $l_{ij}$ is shown in Equation (4):

(4) $$con_{i,j}={\left(1-e^{-\rho_{i,j}}\right)}^{\left|l_{ij}\right|/R}.$$

#### 3.3.3. Heuristic Path Search

Combining the path tree model and connectivity analysis of road sections, the optimal routing path from the source node to the target node is selected using the following criteria. The specific selection criterion for the optimal path is that the path has connectivity above a certain level and is of the shortest length. The selection of the optimal path can be formalized as follows:

(5) $$\begin{array}{cc} \; & \min\sum \left|l_{ij}\right|\cdot g_{ij},\\ \; & s.t.\;con_{ij}\cdot g_{ij}\geq \lambda , \end{array}$$

where the value of the binary variable $g_{ij}$ is 1 when the routing path selects the intersections $o_{i}$ and $o_{j}$; otherwise, the value of $g_{ij}$ is 0, and *s* and *d* are the start and end of the selected segment, respectively. λ is the connectivity threshold of the road section.

### 3.4. Optimal Relay Selection

During the multi-hop transmission of safety messages on the optimal routing path, the optimal relay node is selected at each hop. The HMDR method selects four important metrics for relay selection and analyzes the subjective and objective weights of each metric to obtain a table for the comprehensive utility values of all candidate relays. After excluding the “edge nodes” that are about to leave the communication range, the optimal relay node is selected as the node that forwards the safety message, i.e., the node with the first rank in the comprehensive utility value transmits the message.

To facilitate the description and solution of the problem, it is assumed that *m* vehicles are recorded in the neighbor information table of the current forwarding vehicle and constitute the set of candidate relays $T=\left\{T_{1},T_{2},\cdots ,T_{m}\right\}$; each candidate relay has *n* metrics for evaluating its performance and constitutes the set of evaluation metrics $F=\left\{F_{1},F_{2},\cdots ,F_{n}\right\}$. The current forwarding vehicle updates the metric values of candidate relays in real time and constructs the initial decision evaluation matrix *H* for relay selection after data preprocessing based on related utility functions.

#### 3.4.1. Data Preprocessing

The proposed HDMR method selects four important metrics, i.e., the relative distance and relative speed between the current forwarding vehicle and the candidate relay, the RSS of the candidate relay, and area density, to evaluate the performance of the candidate relay. First, the data need to be preprocessed using the corresponding utility functions for the four detected metrics.

(1) Relative distance

The relative distance between the current forwarding vehicle and the candidate relay is the next hop distance for safety message forwarding. If the relative distance is shorter, the safety message requires more hops to cover the target area or reach the destination. Assuming that the location of the current forwarding vehicle is $\left(x_{0},y_{0}\right)$, the location of its *j*th candidate relay is $\left(x_{j},y_{j}\right)$; then, the relative distance between them can be expressed as follows:

(6) $$\Delta d_{j}=\sqrt{{\left(x_{0}-x_{j}\right)}^{2}-{\left(y_{0}-y_{j}\right)}^{2}},$$

The utility value of the relative distance $\Delta d_{j}$ is as follows:

(7) $$U\left(\Delta d_{j}\right)=\frac{\Delta d_{j}}{R},$$

where *R* is the transmission radius.

(2) Relative speed

The relative speed between the current forwarding vehicle and the candidate relay is another important factor that affects the link stability. The smaller the relative speed, the less likely the candidate relay will easily leave the communication range of the current forwarding vehicle, which also means the higher the link stability. Assuming that the speed of the current forwarding vehicle is $v_{0}$, the speed of the *j*th candidate relay is $v_{j}$, and the direction angle between the two vehicles is $\theta_{0,j}$; the relative speeds of the current forwarding vehicle *i* and the candidate relay *j* can be expressed as follows:

(8) $$\Delta v_{j}=\sqrt{v_{0}^{2}+v_{j}^{2}-2v_{0}v_{j}\cos\left(\theta_{0,j}\right)}.$$

The utility value of the relative speed $\Delta v_{j}$ is:

(9) $$U\left(\Delta v_{j}\right)=\frac{\Delta v_{j}}{2v_{max}},$$

where $v_{max}$ is the maximum speed limit of vehicles.

(3) RSS

The RSS of the candidate relay is a key factor in ensuring whether the safety message can be successfully received or not, and a larger RSS value indicates that the safety message is more likely to be successfully received. The value of RSS depends on the distance between vehicles; the further the distance, the smaller the RSS value. Assuming that the RSS value of the *j*th candidate relay recorded in the neighbor information table of the current forwarding vehicle is $rss_{j}$, the utility value of $rss_{j}$ is as follows:

(10) $$U\left(rss_{j}\right)=\left\{\begin{array}{c} 0,rss_{j}\leq rss_{min},\\ \frac{rss_{j}-rss_{min}}{rss_{max}-rss_{min}},rss_{min}<rss_{j}<rss_{max},\\ 1,rss_{j}\geq rss_{max}, \end{array}\right.$$

where $rss_{min}$ and $rss_{max}$ are the minimum and maximum thresholds of RSS for not being able to receive and guaranteed to receive safety messages, respectively.

(4) Area density

The area density of a candidate relay is the number of neighboring vehicles per unit distance length, which reflects the degree of traffic congestion on the lanes; too small an area density may lead to link interruptions, while too large an area density is more likely to generate message conflicts. Only if the density is appropriate can it be guaranteed that the selected relay will achieve a higher quality of routing. The area density of candidate relay *j* and its utility value are as follows:

(11) $$\rho_{j}=\frac{m}{\pi R^{2}},$$

(12) $$U\left(\rho_{j}\right)=\sin\frac{\pi \cdot \rho_{j}}{\rho_{max}},$$

respectively, where $\rho_{max}$ is the maximum area density.

(5) Decision matrix

The initial evaluation metrics of candidate relays are normalized using the above utility functions, and the initial standardized decision matrix *H* is obtained as follows:

(13) $$H=\left[\begin{array}{cccc} h_{11} & h_{12} & \cdots & h_{1n}\\ h_{21} & h_{22} & \cdots & h_{2n}\\ \vdots & \vdots & \ddots & \vdots \\ h_{m1} & h_{m2} & \cdots & h_{mn} \end{array}\right]={\left(h_{ij}\right)}_{m\times n},$$

where $h_{ij}$ represents the utility value corresponding to the *j*-th evaluation metric of the *i*-th candidate relay.

#### 3.4.2. Relay Evaluation

In this section, we select four evaluation metrics and use a multi-attribute decision-making-based method to evaluate the performance of candidate relays. In order to obtain comprehensive utility values that can more accurately reflect the performance of candidate relays, the subjective and objective weights of the candidate relays are comprehensively considered and calculated based on ordinal relationship analysis and CRITIC weighting methods, respectively.

(1) Subjective weights

Ordinal relationship analysis is an improved Analytic Hierarchy Process (AHP) method without a consistency test, which is adopted to calculate the subjective weights of evaluation metrics of candidate relays in this section.

Assuming that there is an ordinal relationship $f_{1}≻f_{2}≻\cdots ≻f_{n}$ between the *n* evaluation metrics, which indicates that the adjacent evaluation metric $f_{i}$ is more important than the evaluation metric $f_{i-1}$, the ratio of importance between them is denoted as $r_{k}$, and $r_{k}$ is related to the subjective weights of evaluation metrics, as shown below:

(14) $$r_{k}=\frac{w_{sub,k-1}}{w_{sub,k}},$$

where $w_{sub,k-1}$ and $w_{sub,k}$ are the subjective weights of $f_{k-1}$ and $f_{k}$, respectively. $r_{k}$ is illustrated in Table 2. It should be noted that the evaluation metrics involved in this study have the following ordinal relationship: relativedistance≻RSS≻areadensity≻relativespeed.

The subjective weight of metric $f_{n}$ is calculated as follows:

(15) $$w_{sub,n}={\left(1+\sum_{j=2}^{n}\prod_{i=j}^{n}r_{i}\right)}^{-1}.$$

According to the proportional relationship between adjacent evaluation metrics, the subjective weight of metric $f_{j}$ can be derived based on Formula (19), as shown in Equation (20):

(16) $$w_{sub,j}=w_{sub,n}\cdot \prod_{i=j+1}^{n}r_{i}.$$

(2) Objective weights

The CRITIC weighting method is an objective empowerment method based on the volatility and the correlation of data, which is utilized to calculate the objective weights of evaluation metrics of candidate relays in this section.

The volatility of data is expressed in the form of standard deviation. The larger the standard deviation of a metric, the more information it reflects and the more weight it should be assigned. Let $S_{j}$ denote the standard deviation of the *j*-th metric; then, calculate $S_{j}$ as follows:

(17) $$S_{j}=\sqrt{\frac{\sum_{i=1}^{n}\left(h_{ij}-{\bar{h}}_{j}\right)}{n-1}},$$

(18) $${\bar{h}}_{j}=\frac{1}{n}\sum_{i=1}^{n}h_{ij},$$

where ${\bar{h}}_{j}$ is the mean value of the *j*-th metric of candidate relays.

The correlation of data is expressed by the correlation coefficient. The larger the correlation coefficient of a metric, the more it reflects the same information and the less weight it should be assigned. Let $c_{ij}$ represent the correlation coefficient between the *i*-th and *j*-th metrics; then, the correlation coefficient for metric $f_{j}$ is as follows:

(19) $$C_{j}=\sum_{i=1}^{n}\left(1-c_{ij}\right).$$

Considering the above two aspects together, the objective weight of metric $f_{j}$ is calculated as shown below:

(20) $$w_{obj,j}=\frac{S_{j}\cdot C_{j}}{\sum_{i=1}^{n}\left(S_{j}\cdot C_{j}\right)}.$$

(3) Comprehensive weights and utility values

We evaluate the performance of candidate relays from both subjective and objective aspects. After obtaining the subjective and objective weights of the metrics, the adjustment coefficient β (0 < β < 1) is introduced and the simple weighting method is used to calculate the comprehensive weights of metrics. The comprehensive weight of metric $F_{j}$ is as follows:

(21) $$w_{sum,j}=\left(1-\beta \right)\cdot w_{sub,j}+\beta \cdot w_{obj,j}.$$

Similarly, the comprehensive weights of other metrics can be calculated. Finally, the comprehensive utility values of candidate relays are calculated by combing the comprehensive weights and the decision matrix through the simple weighting method, and the comprehensive utility value $C_{i}$ of any candidate relay $q_{i}\left(1\leq i\leq m\right)$ can be obtained, which is calculated as follows:

(22) $$C_{i}=\sum_{j=1}^{n}w_{sum,j}\cdot h_{ij}.$$

Candidate relays with higher comprehensive utility values exhibit better performance, as evidenced by better link quality while maintaining longer one-hop distances.

#### 3.4.3. Relay Selection

Theoretically, selecting the candidate relay with the higher comprehensive utility value can result in greater gains; however, due to the high-speed mobility of vehicles, candidate relays at the edge of the current forwarding vehicle’s communication range may leave that range in a short period of time, resulting in the interruption of the communication link between them and routing failures. Therefore, these “edge” vehicles need to be removed before selecting a relay. Here, the method of the literature [25,26] is utilized to estimate the link duration between vehicles, and a relay selection strategy combining the link duration estimation is proposed.

Suppose that we now need to estimate the link time between vehicles $q_{a}$ and $q_{b}$, the locations of which are $\left(x_{a},y_{a}\right)$ and $\left(x_{b},y_{b}\right)$, the speeds of which are $v_{a}$ and $v_{b}$, and of which the angles of direction are $\theta_{a}$ and $\theta_{b}$, respectively. Then, the remaining connected link time for the two vehicles is as follows:

(23) $$Linktime_{a,b}=\frac{-\left(AB+CD\right)+\sqrt{\left(A^{2}+C^{2}\right)\cdot R^{2}-{\left(AD-BC\right)}^{2}}}{A^{2}+C^{2}},$$

where *A*, *B*, *C*, and *D* are calculated as follows:

(24) $$\begin{array}{cc} \; & A=v_{a}\cos\theta_{a}-v_{b}\cos\theta_{b};\\ \; & B=x_{a}-x_{b};\\ \; & C=v_{a}\sin\theta_{a}-v_{b}\sin\theta_{b};\\ \; & D=y_{a}-y_{b}. \end{array}$$

The current forwarding vehicle $q_{0}$ estimates the link time with all candidate relays based on the above strategy, and if its link time with candidate relay $q_{i}$ is less than the threshold value Δt, i.e., $Linktime_{q_{0},q_{i}}\leq \Delta t$, then the candidate relay $q_{i}$ is removed from the optional relays, thus minimizing the link with the “edge” vehicles, which is conducive to guaranteeing the stability of the communication link. Finally, among the remaining candidate relays that satisfy the link time, the vehicle with the largest integrated utility value is selected as the next hop relay.

## 4. Experiments and Analysis

### 4.1. Experimental Scenario and Parameters

**Figure 7:**
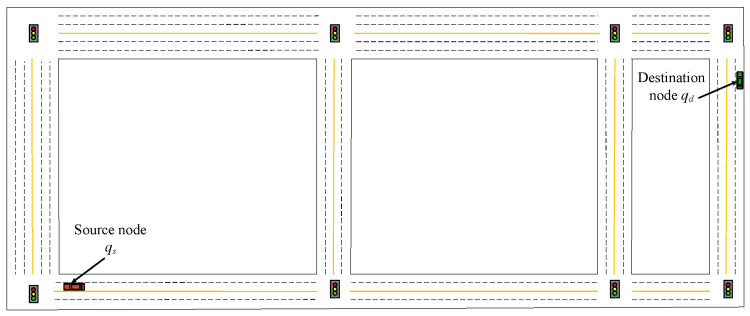
Experimental scenario.

In this section, we implemented and evaluated our proposed HDMR method and comparative methods in a simulated vehicular environment built via MATLAB, an experimental urban traffic scenario with multiple lanes in both directions, as shown in Figure 7. Initially, the vehicles obey Poisson distribution, vehicle $q_{s}$ is the source node of the safety message, vehicle $q_{d}$ is the target node, safety messages are transmitted in multiple hops along the lanes via V2V communication, and the experimental data are processed using Python. The main simulation parameters related to the experiment are shown in Table 3.

### 4.2. Experimental Results and Analysis

#### 4.2.1. Sensitivity Analysis of the β Value

**Figure 8:**
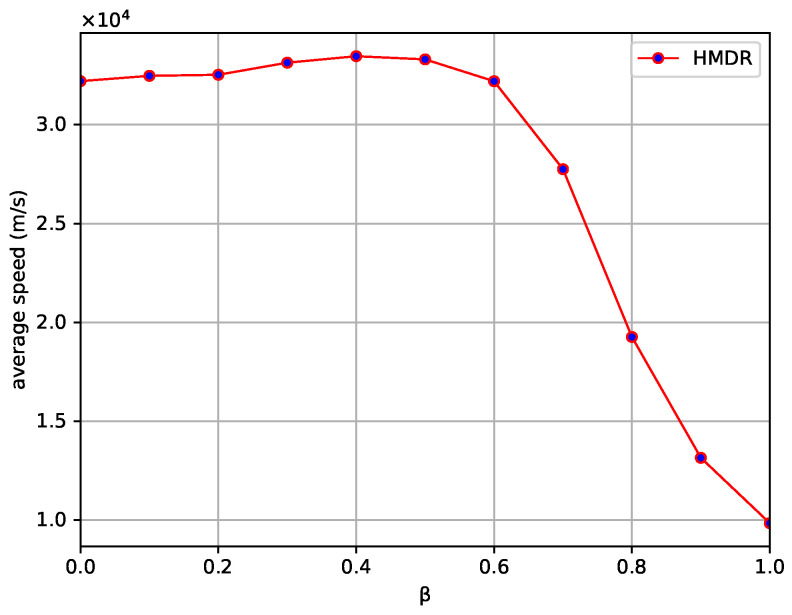
Effect of different β values on the propagation speed of safety messages.

In this study, the subjective and objective weights of four metrics are comprehensively considered in evaluating candidate relays and weighted via the adjustment factor β. Therefore, the value of β is adjusted at intervals of 0.1 under different traffic density conditions, and the changes in the average propagation speed of the safety messages of the proposed HDMR method are observed. As shown in Figure 8, the average propagation speed is positively correlated with the value of β at the beginning, and the speed is the fastest when β exhibits values from 0.3 to 0.5; it then shows a rapidly decreasing trend, so the value of β in the comparison experiments is taken as 0.4.

It can be seen that the subjective weight calculation method based on the ordinal relationship method combined with expert experience can better reflect the performance of candidate relays and effectively improve the performance of HDMR as its proportion increases. The objective weight calculation method based on CRITIC cannot effectively reflect the importance of the evaluation metrics of candidate relays and has difficulty in accurately selecting relays with better overall performance when their proportion is higher; however, it can effectively differentiate between the metrics that have a certain degree of correlation, and when the objective weight obtained based on it is combined with the subjective weight, it can, to a certain extent, improve the accuracy of the evaluation of the candidate relay, which is conducive to the selection of the best relay.

#### 4.2.2. Road Connectivity Analysis

**Figure 9:**
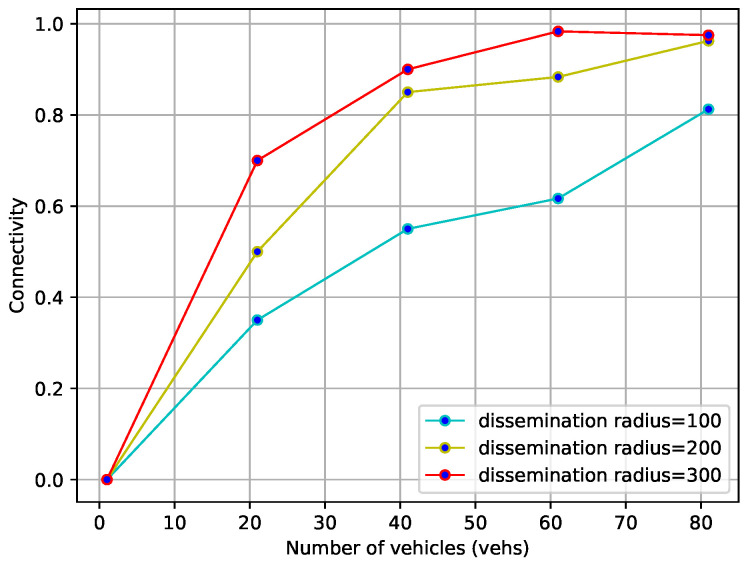
Impact of number of vehicles and transmission radius on connectivity.

From Section 3.3.2, it can be seen that the defined special density determines the connectivity of the road section, which is related to the number of vehicles, the transmission radius, and the length of the road section. This section verifies its rationality through two experimental scenarios. In the first case, the number of vehicles grows from 1 to 100 and the transmission radius is 100, 200, and 300 m. From Figure 9, it can be seen that the connectivity of the road section is proportional to both the number of vehicles and the transmission radius for a certain length of the road section.

**Figure 10:**
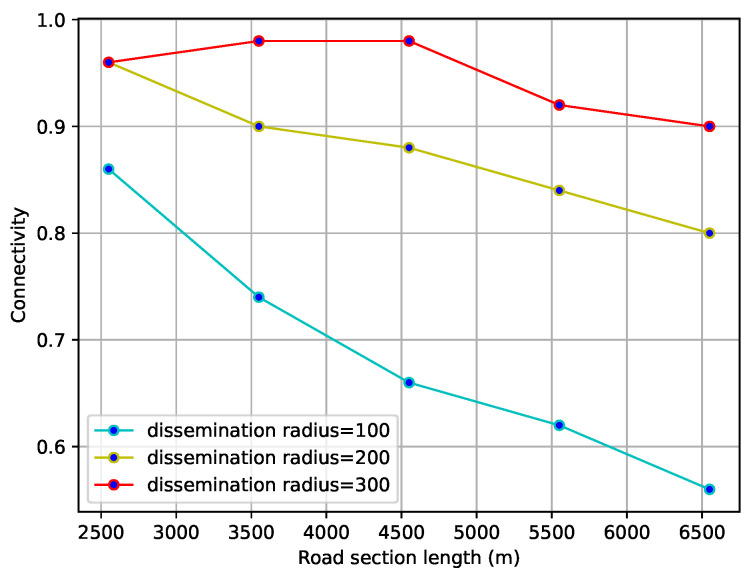
Impact of length of road section and transmission radius on connectivity.

In the second case, the length of the road section ranges from 2500 to 6500 m and the transmission radius is 100, 200, and 300 m. From Figure 10, it can be seen that for a given number of vehicles, the road connectivity is inversely proportional to the length of the road section and directly proportional to the transmission radius. Therefore, the two experimental results described above allow us to verify the justification of the special density defined.

#### 4.2.3. Comparative Results and Analysis

The selection of paths and the selection of relays are the two main factors that affect the performance of routing methods; therefore, several typical methods that can improve the selection strategies of both are selected for comparison and, thus, used to verify the effectiveness of the proposed HMDR. Specifically, four methods, namely, BRAVE [27]; MISR [28]; IGCR [29]; OPBR [30]; and simplified HMDR, which ignores the link time estimation in the optimal relay selection phase, are selected as comparison models in the experimental section. BRAVE uses Dijkstra’s algorithm to find the shortest routing path and a distance-based random back-off time to select the relay. MISR selects routing paths taking into account the connectivity of road segments and gives preference to wider paths; it selects relays based on distance and link quality. IGCR also considers the connectivity of road segments when selecting routing paths, particularly in relation to vehicle mobility, direction, and traffic density. In addition, it selects vehicles that are closer to the destination and faster as relays. OPBR determines the routing path by continuously selecting relays; specifically, it selects vehicles as relays that are closer to the destination, not obscured by buildings, and maintain the communication link.

**Figure 11:**
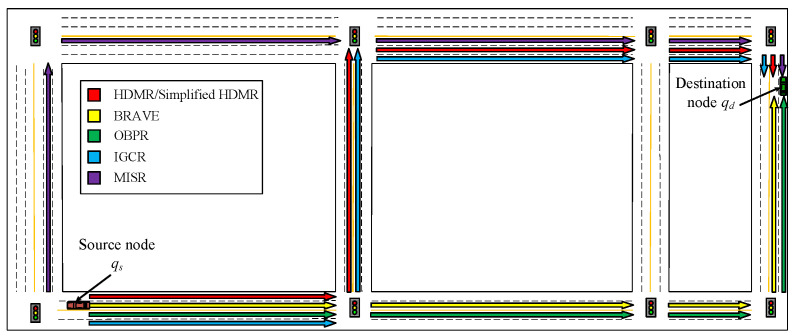
Routing paths of methods.

Figure 11 shows the routing paths of all the methods in the experimental scenario, and it can be seen that they select different routing paths due to the differences in path selection strategies. In short, BRAVE and OPBR tend to select the shortest paths, MISR tends to select wider paths with guaranteed connectivity, and our proposed method and IGCR tend to select paths with higher connectivity of road segments. Then, several performance metrics of the six methods are compared by determining the average value through multiple experiments under different traffic densities.

**Figure 12:**
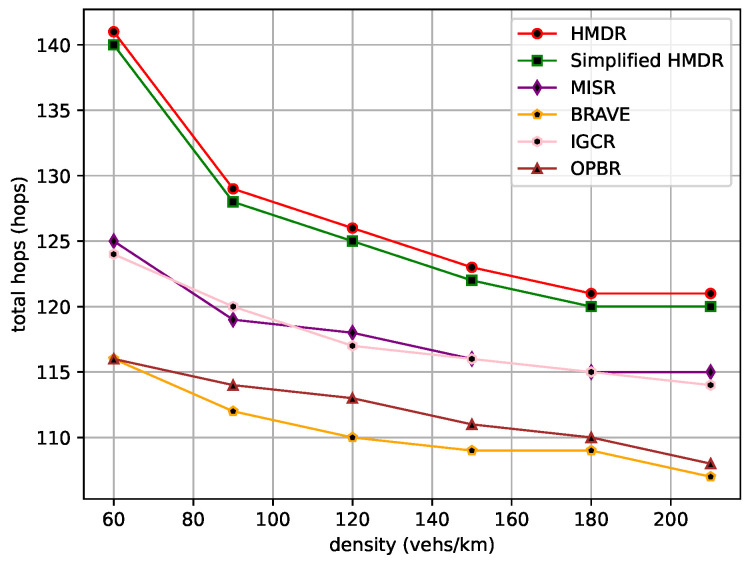
Total hops under different traffic densities.

**Figure 13:**
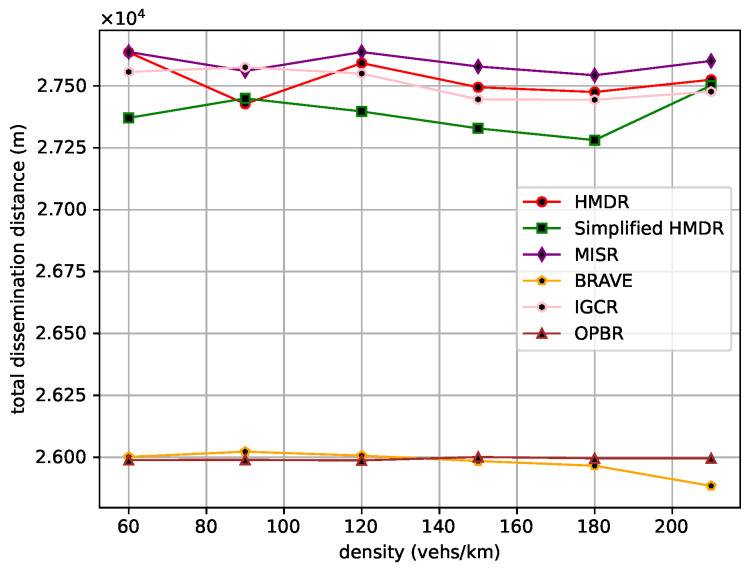
Total transmission distance under different traffic densities.

**Figure 14:**
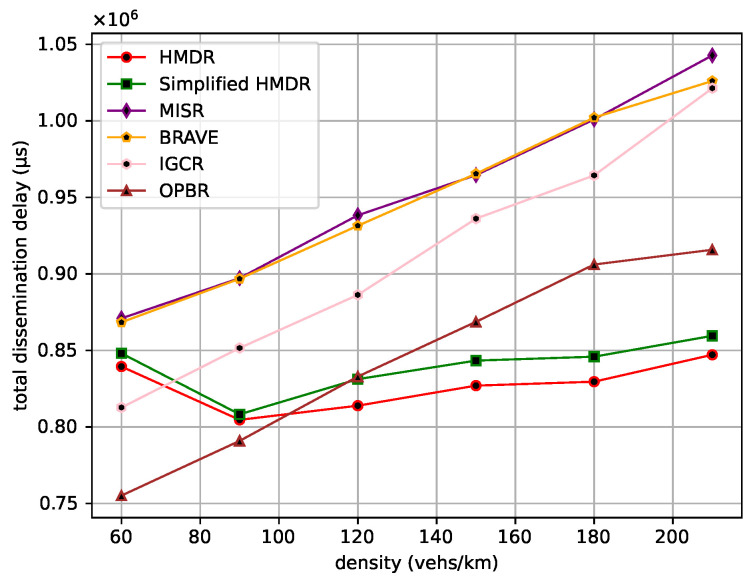
Total transmission delay under different traffic densities.

The transmission of safety messages is a multi-hop forwarding process; first of all, the total hops, the total transmission distance, and the total transmission delay of the safety message from the source node to the destination node are compared under different traffic density conditions. The comparison results are shown in Figure 12, Figure 13 and Figure 14.

BRAVE always selects the shortest routing path as it uses Dijkstra’s algorithm, and OPBR is also able to find the shortest path in our experimental scenario by continuously selecting relays. Both of them try to select a candidate relay with a longer distance within the one-hop range; thus, they have smaller total hops and transmission distances. By considering the connectivity of road segments, the other four methods select relatively long routing paths. In addition, compared with MISR and IGCR, our proposed HMDR and simplified HMDR methods further enhance the transmission reliability at the expense of reducing the one-hop distance, which is beneficial to reducing transmission delay, despite having the maximum number of hops. Specifically, the total number of hops for the proposed HMDR method is similar to that of the simplified HMDR method, while it is approximately 10.00% to 21.55% more than BRAVE and OPBR and approximately 5.22% to 13.71% more than MISR and IGCR. Moreover, the total transmission distance of HMDR is also similar to that of the simplified HMDR, MISR, and IGCR, while it is approximately 5.40% to 6.34% more than BRAVE and OPBR. Moreover, as can be seen in Figure 14, HDMR performs slightly worse than IGCR and OPBR in the beginning, which is due to our selection strategy sacrificing a larger one-hop distance to guarantee the connectivity of road segments, and this strategy does not bring as much benefit as selecting a longer one-hop distance relay when the traffic density is small. When the traffic density increases, the advantage of HDMR is demonstrated. Particularly, at higher traffic densities, HDMR reduces the total transmission delay by about 7.49% compared to the better-performing OPBR and by about 17.05% to 18.76% compared to the three methods, namely, BRAVE, MISR, and IGCR. Due to the lack of link time estimation, simplified HMDR still has a small probability of selecting “edge” vehicles and, therefore, experiences lower performance than HMDR.

**Figure 15:**
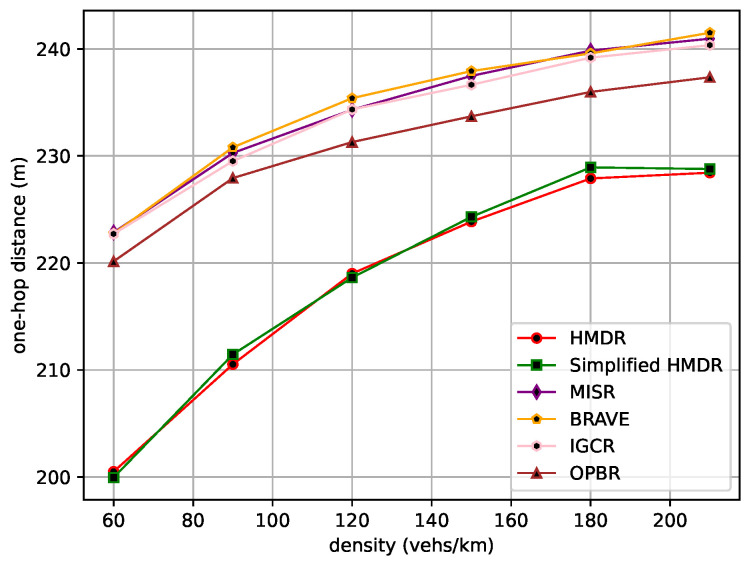
Average one-hop distance under different traffic densities.

**Figure 16:**
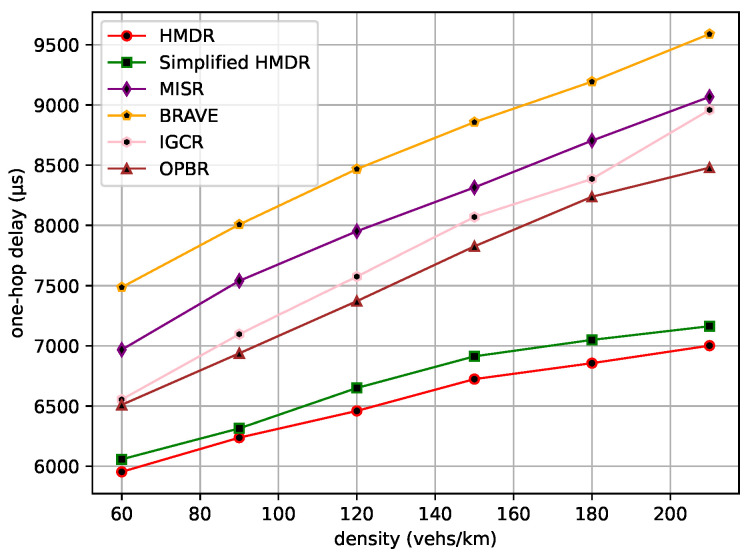
Average one-hop delay under different traffic densities.

**Figure 17:**
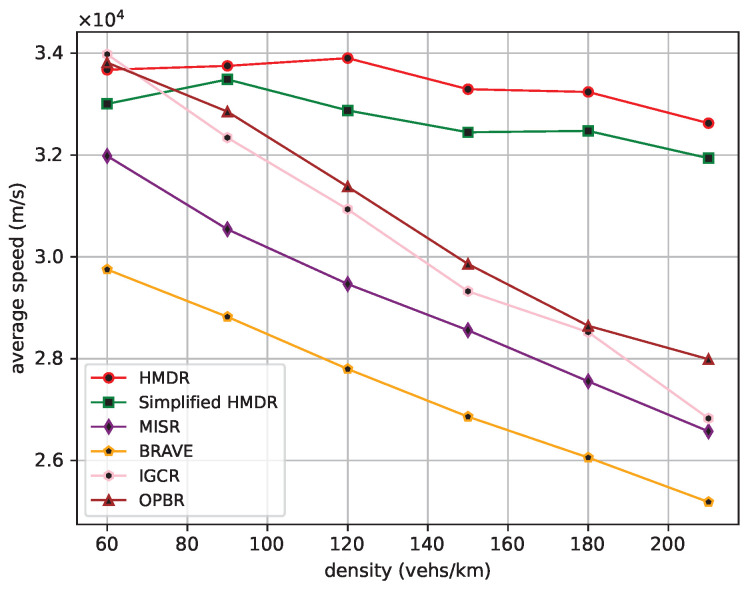
Average transmission speed under different traffic densities.

The average one-hop distance, the average one-hop delay, and the average speed of the safety message from the source node to the destination node are then compared under different traffic density conditions. The comparison results are shown in Figure 15, Figure 16 and Figure 17.

As traffic density increases, the average one-hop distance increases for all methods. Different from other methods, our proposed HMDR and simplified HMDR simultaneously consider multiple evaluation metrics when selecting relays and use a combination of subjective and objective methods to evaluate the comprehensive weights of candidate relays; thus, the link stability is higher, although the one-hop distance of the selected relay is shorter. The other methods also simultaneously consider multiple factors to select a path but lack a predictive method for link stability and, therefore, have a higher probability of link interruption. Specifically, the average one-hop distance and one-hop delay of HMDR are similar to simplified HMDR; the average one-hop distance is approximately 3.43% to 10.03% shorter than the other methods, and the average one-hop delay of HMDR is approximately 8.55% to 26.98% lower than the other methods. Furthermore, as traffic density increases, the average transmission speed of each method decreases, which is mainly due to the increase in transmission delay. As can be seen in Figure 17, HDMR performs comparably to IGCR and OPBR in sparse traffic. However, since the average one-hop delay of our proposed method has the slowest upward trend, it can always transmit at a high speed. Furthermore, when the traffic density increases, the advantage of our method is gradually highlighted, and the average transmission speed of our method is 16.56% to 29.54% faster than the other methods when in dense traffic scenarios.

## 5. Conclusions

Currently, urban traffic safety is an important research topic, and safety message routing for vehicular networks is of great research interest and application value. The V2V-based routing method is more suitable for application in urban scenarios and does not have to rely on the construction of infrastructure, such as RSUs, reflecting its advantages of low latency and low cost. In this study, we proposed a vehicular safety message routing method HMDR that combines path search and relay selection for timely and reliable transmission in intricate traffic scenarios. It uses a heuristic path search method based on road connectivity to select the optimal global routing path to avoid the local optimal problem of path searching. It selects the optimal relay node based on multi-attribute decision-making to accurately evaluate the relay performance in intricate traffic scenarios. The experimental results show that the proposed HMDR tends to choose the path with better connectivity and shorter length, with the characteristics of a lower delay and a higher speed, which is superior to other methods. Therefore, it can be said that HMDR helps to improve the performance of vehicular safety message transmission in intricate traffic scenarios and provides timely data support for secure driving.

However, V2V communication-based routing methods face the problem of poor road connectivity when in sparse traffic and message collisions when in dense traffic, which is less suitable for long-distance transmission in urban scenarios; while V2I-based communication effectively improves communication quality and transmission distance, V2I-assisted safety message routing methods will be studied in future work.

## Figures and Tables

**Table 1:** The Meaning of the Main Variables.

Variable	Description
$o_{i}$	$O=\left\{o_{1},o_{2},\cdots ,o_{i}\right\}$ denotes road intersections, $o_{s}$ is the source, and $o_{d1}$ and $o_{d2}$ are the targets.
$l_{i,j}$, $\left|l_{i,j}\right|$	The road section that joins two intersections $o_{i},o_{j}\in O$, and its length is $\left|l_{i,j}\right|$
$q_{j}$	$Q=\left\{q_{1},q_{2},\cdots ,q_{m}\right\}$ denotes the candidate relays of the current forwarding vehicle.
$f_{j}$	$F=\left\{f_{1},f_{2},\cdots ,f_{n}\right\}$ denotes the evaluation metrics of candidate relays.
$\left(x_{0},y_{0}\right)$	The location of the current forwarding vehicle.
$\left(x_{j},y_{j}\right)$	The location of $q_{j}$.
*R*	The transmission radius of the vehicles.
$v_{0}$	The speed of the current forwarding vehicle.
$v_{j}$	The speed of $q_{j}$.
$v_{max}$	The maximum speed limit of vehicles.
$\theta_{0,j}$	The direction angle between the current forwarding vehicle and $q_{j}$.
$rss_{j}$	The RRS value of $q_{j}$ recorded by the current forwarding vehicle.
$rss_{min}$, $rss_{max}$	The minimum and maximum thresholds of the RRS value.
$\rho_{j}$	The area density of $q_{j}$.
$\rho_{max}$	The maximum area density of vehicles.
$con_{i,j}$	The connectivity of $l_{i,j}$.

**Table 2:** Description of the values of $r_{k}$.

The Value of $r_{k}$	Description
1.0	$f_{k-1}$ is as equally important as $f_{k}$.
2.0	$f_{k-1}$ is slightly more important than $f_{k}$.
3.0	$f_{k-1}$ is significantly more important than $f_{k}$.
4.0	$f_{k-1}$ is more important than $f_{k}$.
5.0	$f_{k-1}$ is extremely more important than $f_{k}$.

**Table 3:** Main simulation parameters.

Parameter	Value
Transmission radius R/m	270
Vehicle speed ${v/\mathrm{ms}}^{-1}$	8∼16
Maximum vehicle density $\rho_{max}/\mathrm{vehs}\;\mathrm{per}\;\mathrm{km}$	250
Minimum RSS threshold $rss_{min}/\mathrm{dBm}$	−85
Beacon sending interval/s	1
Minimum distance between vehicles/m	1
The value of β	0.4

## Data Availability

Data are contained within the article.

## Abbreviations

The following abbreviations are used in this manuscript:

HDMR	Heuristic path search and multi-attribute decision-making-based routing method
RSS	Received signal strength
CRITIC	Criteria Importance through Intercriteria Correlation
VANET	Vehicular Ad Hoc Network
V2V	Vehicle-to-vehicle
V2I	Vehicle-to-infrastructure
